# Mucosal Fenestration Closure: A Case Report on Regenerative Therapeutic Strategies

**DOI:** 10.1002/ccr3.70334

**Published:** 2025-03-17

**Authors:** Nabin Thapa, Bibek Kattel, Abhishek Kumar, Sajeev Shrestha

**Affiliations:** ^1^ Department of Periodontology and Oral Implantology BP Koirala Institute of Health Sciences Dharan Nepal; ^2^ College of Dental Surgery BP Koirala Institute of Health Sciences Dharan Nepal

**Keywords:** bone grafting, fenestration, oral mucosa, periodontal debridement, periodontal guided tissue regeneration

## Abstract

Mucosal fenestration, a condition that poses risks to dental integrity, requires effective management to prevent complications such as unwanted tooth loss. The integration of guided tissue regeneration (GTR), Platelet‐rich fibrin (PRF), and bone grafts is an effective strategy for managing mucosal fenestration, ensuring optimal tissue regeneration and long‐term success.

## Graphical abstract

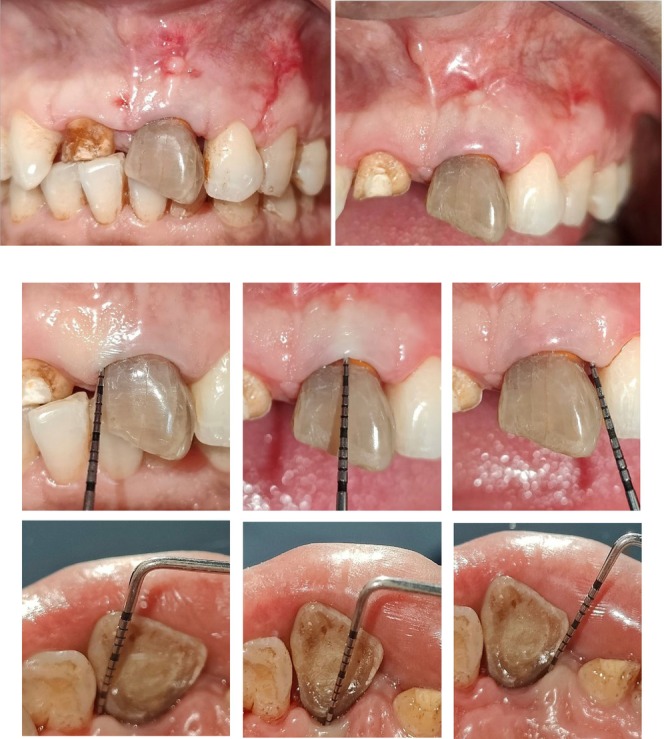

## 1 Introduction

Mucosal fenestration is a specific type of fenestration characterized by a perforated alveolar bone plate and denuded gingiva and mucosa leading to direct exposure of the root surface [1, 2]. Although the exact cause is not fully understood, several factors are believed to contribute to its occurrence, including overlying gingival biotype, buccally placed root, occlusal factor, orthodontic tooth movement, and associated chronic peri‐apical and periodontal infection [3, 4]. These lesions are often subtle and lack noticeable symptoms, leading to reduced patient awareness and delayed treatment.

Additionally, they may arise in situations that are difficult to detect or assess with standard dental X‐rays [4]. This can result in challenges such as poor plaque management, increased tooth root sensitivity, root decay, and cosmetic concerns, complicating the decision between tooth extraction and preservation [5]. This article describes the case of mucosal fenestration due to a chronic periapical infection caused by trauma, highlighting a management approach that included regenerative periodontal surgery with the use of a guided tissue regeneration (GTR) membrane, platelet‐rich fibrin (PRF), and bone grafts.

## 2 Case Presentation

### 2.1 Patient History and Clinical Presentation

A 40‐year‐old male patient was referred to the Department of Periodontology and Oral Implantology with concerns regarding a discolored upper front tooth accompanied by a hole in the gum, a history of trauma to the upper anterior region of the upper jaw, and a history of hypertension for 10 years and has been taking amlodipine. The habit history is noncontributory. For reasons unknown, the patient did not seek active treatment but had undergone Root canal therapy a year back as he presented to the outdoor patient department for discoloration of the offending tooth.

**FIGURE 1:**
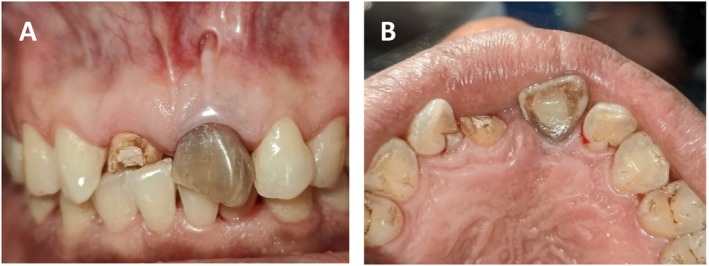
(A, B) Intra‐oral photograph after non‐surgical therapy showing presence of mucosal fenestration along with the discoloration and normal palatal tissue with respect to tooth number‐9.

Intraoral examination revealed a discolored clinical crown of tooth number 9 (Universal system of numbering of teeth) with class I composite restoration with fractured (tooth number‐8 and 9) Ellis class III [6], Millers Grade I mobility [7] with normal probing depth (Figure 1A,B). Additionally, a mucosal fenestration of size 5 × 3 mm^2^ filled with debris at the periapical area was noted. Palatal tissue appeared within normal limits. There was no suppuration or tenderness noted.

### 2.2 Investigations

**FIGURE 2:**
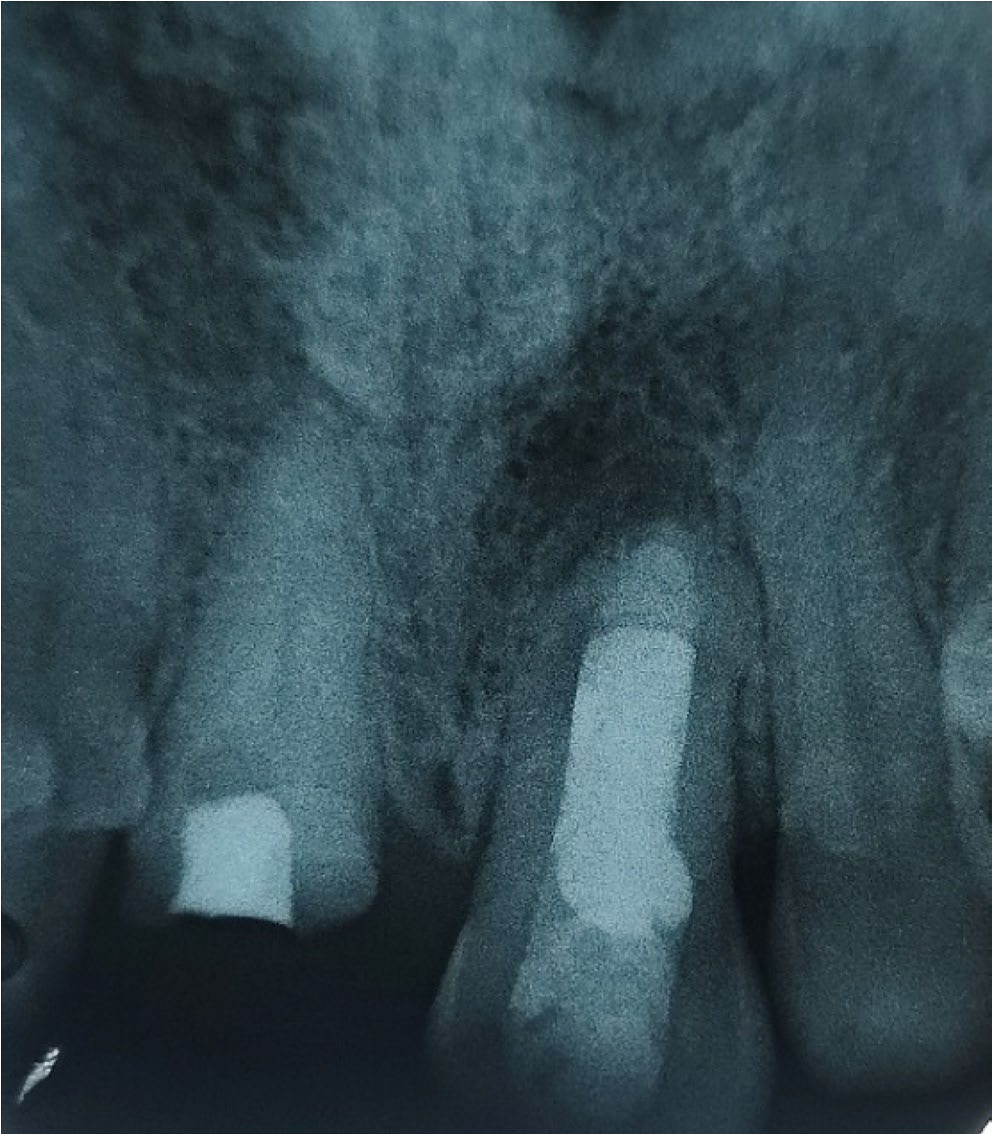
Pre‐operative radiograph showing presence of apical radiolucency.

Vitality test yielded unresponsive results leading to the Intraoral periapical radiograph (Figure 2) revealing the history of root canal therapy, a periapical radiolucency of size 3 × 4 mm^2^, with intact interdental bones. Considering all the findings, a diagnosis of mucosal fenestration was made. As the prognosis was not deemed hopeless and there were no absolute indications for extraction, coupled with the patient's strong desire to preserve the tooth, we decided on the following conservative treatment approach rather than proceeding with extraction.

### 2.3 Treatment

**FIGURE 3:**
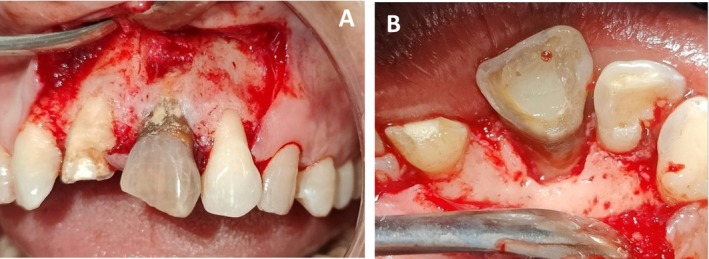
Intra‐oral photographs post flap reflection–(A): Periapical extrusion of sealer material and persistent periapical infection and (B): Palatal view.

**FIGURE 4:**
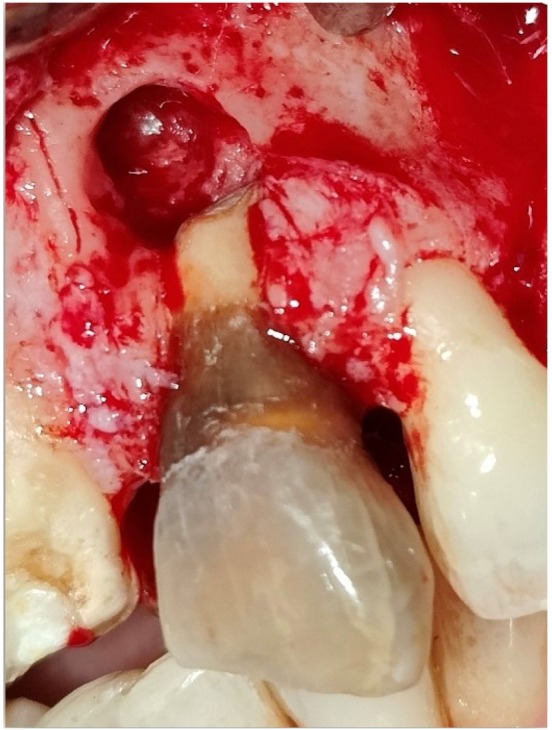
Intraoperative post‐debridement photograph showing presence of bony defect at apical region.

**FIGURE 5:**
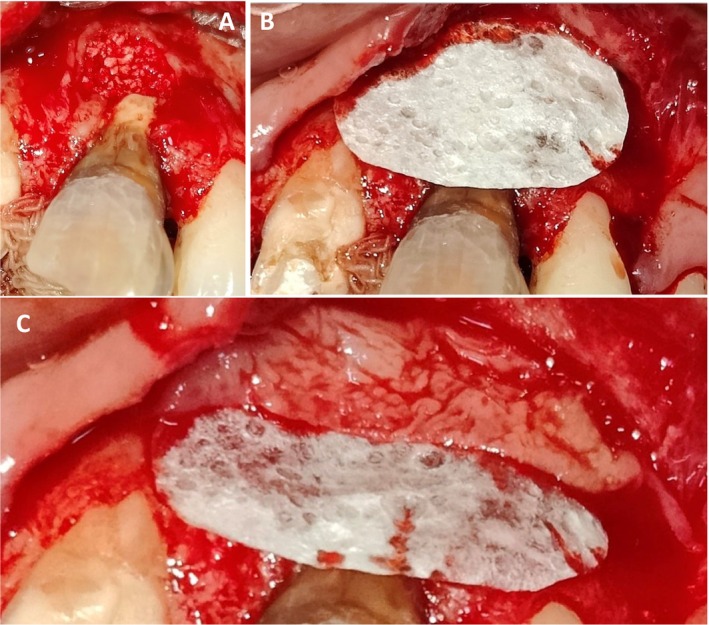
Intra‐operative photographs showing—(A): Placement of porous bony graft. (B): Placement of GTR membrane. (C): Placement PRF Membrane.

**FIGURE 6:**
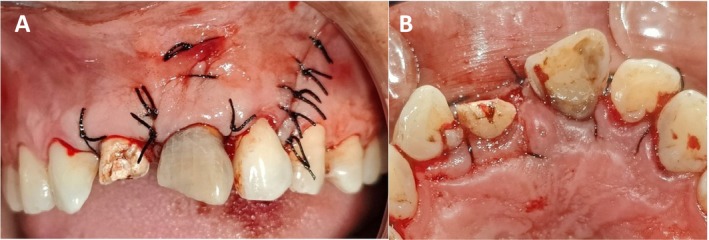
Intra‐operative photograph showing primary closure with 4–0 silk suture. (A): Buccal view (B): Palatal view.

Informed consent was taken, and non‐surgical treatment scaling and deep surface debridement was performed initially. The apicectomy followed by guided tissue regeneration was planned. In subsequent follow‐ups, anterior superior alveolar and nasopalatine nerve blocks were given with 2% lidocaine with 1:200000 epinephrine. A crestal incision and vertical releasing incision were given, and a full thickness mucoperiosteal flap was raised beyond the mucogingival line to give access to the root apex. Periapical extrusion of sealer material and persistent periapical infection were seen in the periapical area of 9 (Figure 3A,B). Root surface debridement and curettage of periapical bone were performed in the periapical area of 9 (Figure 4) apicectomy was done, and then the Root canal was sealed with glass ionomer apically. The debrided area was cleaned, and a sterile bioresorbable demineralized bone matrix (DMBM—Xenograft) (Figure 5A) was placed at the apical area, followed by a GTR membrane (Type 2 Collagen of fish origin) (Figure 5B) followed by platelet‐rich fibrin prepared by following choukroun's protocol [8] in the form of a 1 mm thick membrane (Figure 5C) The flap was then re‐approximated with a 4–0 silk suture (Figure 6A,B) and was advised to take antibiotics: Amoxicillin 500 mg + clavulanate 125 mg three times per day for 5 day for the broad‐spectrum activity against a wider range of bacteria because of prolonged surgical duration, extensive flap reflection, and significant bone scrapping, where the risk of infection is elevated due to increased tissue manipulation and exposure. Analgesic Ibuprofen 400 mg three times per day for 3 day was added to the regimen. Postoperatively, the patient was asked to rinse the mouth with 0.2% chlorhexidine after 24 h. In order to prevent further stress on the treated area, the tooth was taken out of occlusion, and the patient was advised to maintain optimal oral hygiene, use the Charter brushing technique for gentle and effective cleaning, and refrain from biting hard foods directly with the anterior teeth.

## 3 Outcome and Follow‐Up

**FIGURE 7:**
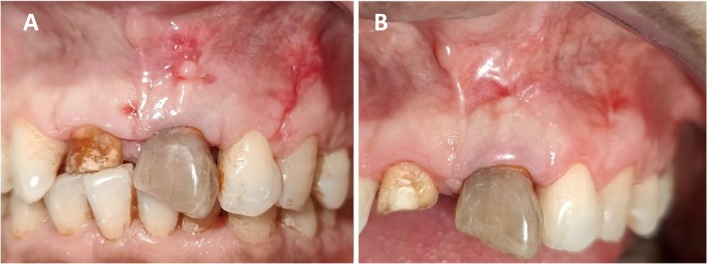
(A) Intra‐oral photograph after suture removal. (B): 3 months follow‐up showing satisfactory healing.

**FIGURE 8:**
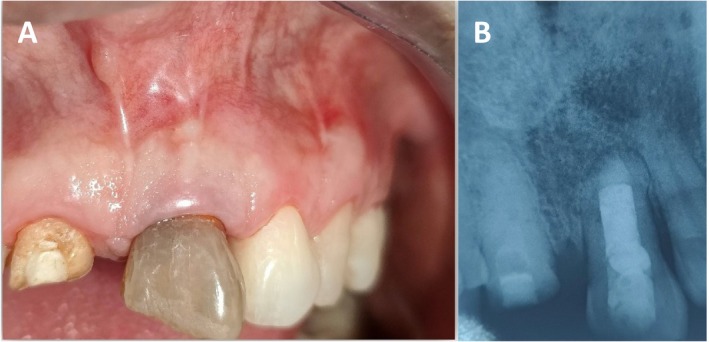
(A, B) Intra‐oral photograph and IOPA radiograph showing after 4 months follow‐up showing satisfactory healing of mucosal and periapical tissue clinically and radiographically.

**FIGURE 9:**
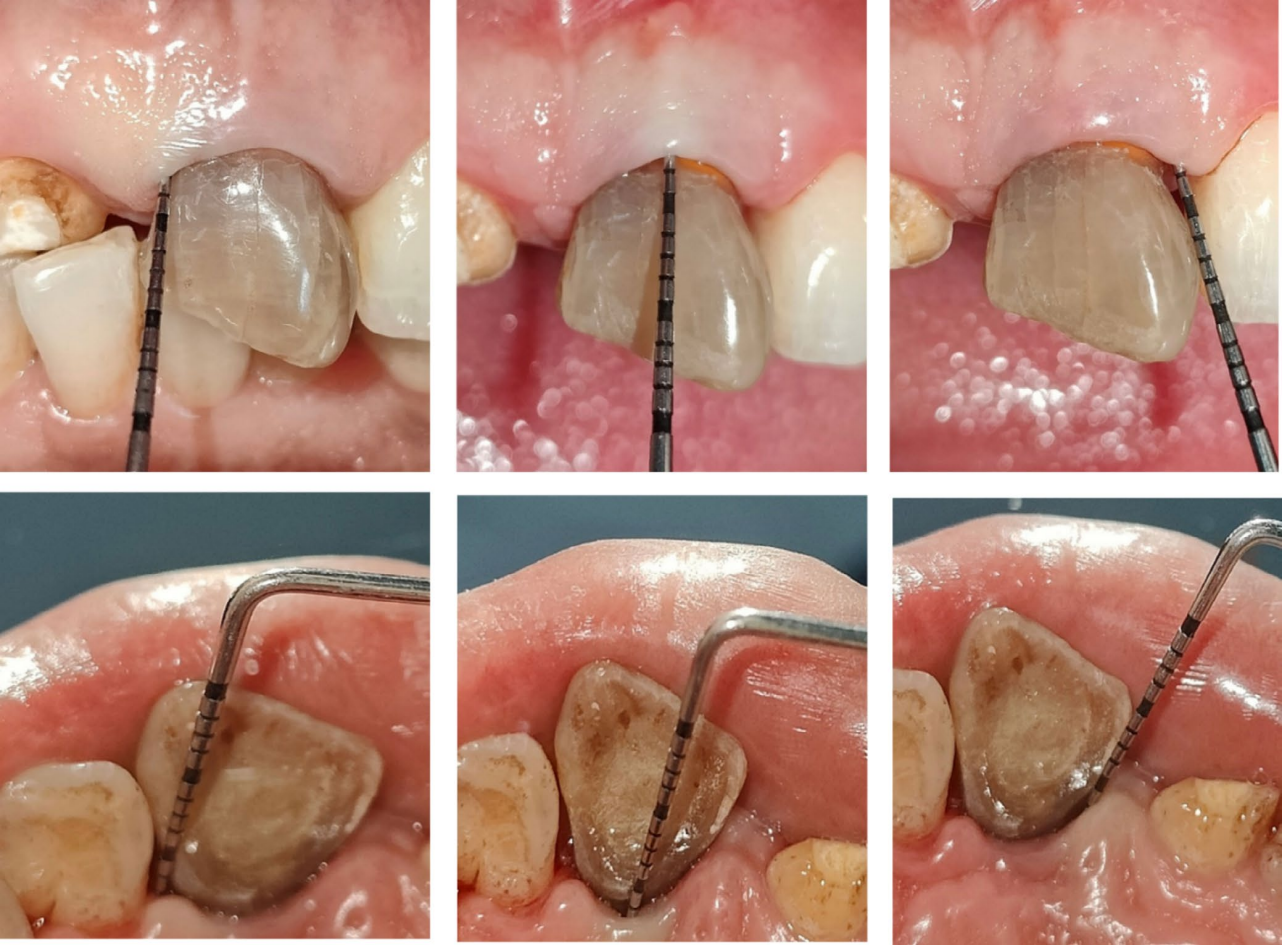
Intra‐oral photograph showing decreased probing depth after treatment.

The suture was removed after 2 weeks, and clinical examination revealed the healing was satisfactory (Figure 7) Then subsequent follow‐up was maintained in 1 month, 3 months (Figure 7B) and 4 months. (Figure 8) Subsequent follow‐up revealed complete closure of fenestration, with optimal apical healing ensured clinically with examination and the decreased probing depth. (Figure 9) Radiographically, the healing process was evident (Figure 8B) affirming the effective management of the condition.

## 4 Discussion

Gingival and mucosal fenestrations, along with dehiscence, are rare entities that, whenever present, pose significant challenges for clinicians. Not all fenestrations require surgical intervention; some cases have been reported where spontaneous closure occurred following oral prophylaxis and improved oral hygiene practices [2, 9]. There are various non‐surgical and surgical procedures documented for managing fenestration. Even when surgical intervention is necessary, non‐surgical therapy remains crucial. It helps to reduce inflammation, facilitates plaque control, and aids in the proper attachment of the surgical flap to the exposed root surface, thereby facilitating fenestration closure [1, 10]. The present case report describes a rare situation where a gingival fenestration was noted in the maxillary left central incisor, which was effectively managed with a combination of apicectomy and a regenerative approach to repair the fenestration defect.

When the apical portion of the tooth is affected in cases of mucosal fenestration, a joint approach of surgical treatment and endodontics may be beneficial. This enables efficient management of the exposed root, addressing both the underlying infection or pathology and restoring the integrity of the surrounding tissues. By integrating these disciplines, the treatment ensures comprehensive healing and reduces the risk of recurrence or complications. A regenerative procedure using guided tissue regeneration and bone graft is easier to perform than a mucogingival procedure, with the added advantage of avoiding trauma of a secondary surgical site for graft harvesting [11]. Limited literature addresses failures in repairing bony fenestrations with repeated soft tissue grafts.

Since soft tissue grafts depend on the underlying bone for support, vascularity, and nutrient supply, bone defects increase the risk of graft failure. In our case, instead of depending only on mucogingival procedures, we chose to use a combination of platelet‐rich fibrin (PRF), porous bone grafts, and guided tissue regeneration (GTR) membranes because of the underlying bone defect. This approach ensures better healing and graft stability [2, 12, 13].

Though the literature on the management of mucosal fenestration is very scarce, some reports available provide valuable insights. The successful management of mucosal fenestration largely depends on accurately identifying its underlying cause. Peacock et al. [14] reported the effective use of connective tissue graft (CTG) in managing gingival fenestration successfully. Chen et al. [2] reported successful management of mucosal fenestrations using demineralized freeze‐dried bone allograft packed into the bony defect and covered with CTG to achieve soft tissue coverage. Mellonig [15] and Flemming et al. [16] demonstrated favorable outcomes with the use of bone graft. Pecora et al. [17] showed that GTR procedures yield superior quality and quantity of regenerated bone for large periapical lesions, with human histological studies confirming that the new tissue formed through GTR therapy consists of mature lamellar bone. GTR techniques enhance the outcomes of bone regeneration following surgical endodontic treatment of teeth with large periapical lesions. Additionally, Travassos et al. [18] reported a successful multidisciplinary approach for managing a fenestration‐type defect. The treatment involved using a GTR membrane and a connective tissue graft after apicectomy. A second surgical intervention was performed to close the remaining fenestration defect with another connective tissue graft and a double papilla flap. Rajula et al. [19] successfully managed a case of mucosal fenestration using a combination of bioactive glass with platelet‐rich fibrin and a free connective tissue graft. Balasubramanian et al. [20] managed a rare presentation involving concomitant gingival recession with an isolated mucosal fenestration using an allograft matrix. Chandra et al. [21] compared PRF‐treated sites to the gold standard CTG, finding that it offered better patient acceptance and a less invasive approach in terms of graft procurement. The study reported less patient discomfort with PRF compared to CTG at both day 10 and 1 month post‐operatively. Additionally, PRF provided the advantages of being less invasive, not requiring an additional donor site, and resulting in quicker wound healing and an early reduction in post‐surgical edema.

Platelet‐Rich Fibrin (PRF) is an autologous fibrin matrix rich in platelets, leukocytes, cytokines, and several growth factors. It was introduced as a safer and simpler alternative to Platelet‐Rich Plasma (PRP) and is a second‐generation platelet concentrate used for its ability to enhance tissue repair and regeneration. With its composition rich in growth factors and possessing cicatricial properties, PRF has shown immense potential in clinical applications, especially in root coverage procedures. PRF acts like a reservoir for active biochemicals, releasing them at a very slow rate and thereby maintaining the stability of grafted biomaterials in their respective positions. This not only reduces the volume of graft material required but also provides an optimal connection between bone particles, enhancing overall graft integration and tissue healing [22, 23, 24].

Soft tissue graft techniques can be effective for covering such fenestration defects. Depending on the conditions at both the donor and recipient sites, various soft tissue graft techniques; such as the double papilla flap, laterally positioned flap, coronally advanced flap, connective tissue graft, and free gingival graft can be applied to manage the defect. A similar approach yielded positive result in a case reported by Pradhan et al. [5] For the treatment of apical lesions associated with mucosal fenestration, optimal root canal treatment and effective retrograde filling to seal the canals are mandatory. Mineral Trioxide Aggregate (MTA) has excellent sealing ability, reliable healing rate, and predictable results. Some literatures also suggested Glass ionomer cements being fast setting than MTA and with ability of forming chemical bond with dentin used as retrograde filling material as a cheaper alternative if adequate isolation is maintained. The glass ionomer cements of newer generation are less affected by moisture and are suitable for clinical use as retrograde filling material [25, 26].

Successful outcomes also depend on thorough osseous defect debridement, GTR procedures, and connective tissue grafting or PRF membrane [21, 27].

In managing mucosal fenestrations, satisfactory outcomes can be achieved by adhering to the established periodontal surgical principles of thorough debridement, proper hard tissue management, and securing primary tissue closure. Careful case selection and proper treatment planning are influenced by many factors such as tooth factors, tooth's strategic importance, bone quality, soft tissue quality and quantity, accessibility to the treatment area, periodontal status around the remaining root, patient's systemic health status, the clinician's expertise and importantly the patient's self‐motivation and adherence to maintenance [28].

In conclusion, mucosal fenestrations and dehiscence are rare entities, but whenever present, they pose significant challenges for clinicians. Large fenestration defects pose further challenges and often result in a poor prognosis. A variety of non‐surgical and surgical procedures have been documented for their management. The treatment regimen described in this case report demonstrated substantial clinical improvement and complete coverage of the mucosal fenestration defect. The combined use of platelet‐rich fibrin (PRF), porous bone grafts, and guided tissue regeneration (GTR) membranes demonstrates a successful and effective approach. Comprehensive debridement, precise bone management, and proper tissue closure are essential for achieving optimal healing and preventing complications. More cases need to be reported to validate the success and reliability of this approach.

## Author Contributions

**Nabin Thapa:** conceptualization, investigation, project administration, writing – original draft. **Bibek Kattel:** investigation, software, writing – original draft. **Abhishek Kumar:** investigation, software, writing – original draft. **Sajeev Shrestha:** project administration, writing – review and editing.

## Consent

Written informed consent was obtained from the patient to publish this report in accordance with the journal's patient consent policy.

## Conflicts of Interest

The authors declare no conflicts of interest.

## Data Availability

The data supporting the findings of the present study are available from corresponding author upon request.

## References

[1] H. M. Jhaveri , S. Amberkar , L. Galav , V. L. Deshmukh , and S. Aggarwal , “Management of Mucosal Fenestrations by Interdisciplinary Approach: A Report of Three Cases,” Journal of Endodontia 36, no. 1 (2010): 164–168, 10.1016/j.joen.2009.06.012. 20003959

[2] G. Chen , C. T. Fang , and C. Tong , “The Management of Mucosal Fenestration: A Report of Two Cases,” International Endodontic Journal 42, no. 2 (2009): 156–164, 10.1111/j.1365-2591.2008.01463.x. 19134044

[3] C. C. Tseng , Y. H. Chen , C. C. Huang , and G. M. Bowers , “Correction of a Large Periradicular Lesion and Mucosal Defect Using Combined Endodontic and Periodontal Therapy: A Case Report,” International Journal of Periodontics and Restorative Dentistry 15, no. 4 (1995): 377–383. 8593987

[4] V. R. Nimigean , V. Nimigean , M. A. Bencze , N. Dimcevici‐Poesina , R. Cergan , and S. Moraru , “Alveolar Bone Dehiscences and Fenestrations: An Anatomical Study and Review,” Romanian Journal of Morphology and Embryology 50, no. 3 (2009): 391–397. 19690764

[5] A. Pradhan , S. Aryal , N. Joshi , and S. Shrestha , “Gingival Fenestration: A Multidisciplinary Approach,” Journal of Nepalese Society of Periodontology and Oral Implantology 3, no. 2 (2019): 78–80, 10.3126/jnspoi.v3i2.30889.

[6] R. G. Ellis , The Classification and Treatment of Injuries to the Teeth of Children, 4th ed. (Year Book Publisher, 1961), 1–229.

[7] S. C. Miller , Textbook of Periodontia, 1st ed. (Blakiston, 1938).

[8] D. M. Dohan , J. Choukroun , A. Diss , et al., “Platelet‐Rich Fibrin (PRF): A Second‐Generation Platelet Concentrate. Part I: Technological Concepts and Evolution,” Oral Surgery, Oral Medicine, Oral Pathology, Oral Radiology, and Endodontics 101, no. 3 (2006): e37–e44, 10.1016/j.tripleo.2005.07.008. 16504849

[9] L. A. Santos‐Pinto , N. S. Seale , A. K. Reddy , and R. C. Cordeiro , “Fenestration Gingival Defect in Erupting Permanent Mandibular Incisors: A Case Report,” Quintessence International 29, no. 4 (1998): 239–242. 9643262

[10] B. G. Askenas , H. R. Fry , and J. W. Davis , “Cervical Enamel Projection With Gingival Fenestration in a Maxillary Central Incisor: Report of a Case,” Quintessence International 23, no. 2 (1992): 103–107. 1641450

[11] P. Gandi , N. Anumala , A. Reddy , and R. Viswa Chandra , “A Regenerative Approach Towards Mucosal Fenestration Closure,” BML Case Reports 2013 (2013): bcr2013009604, 10.1136/bcr-2013-009604. PMC3702851 23749826

[12] T. Prasanth , S. Manandhar , T. S. Satisha , N. Gupta , and P. Kumar , “Revisiting Failures in Mucogingival Surgery,” Medical Journal, Armed Forces India 80, no. 4 (2024): 482–487, 10.1016/j.mjafi.2022.04.007. 39071761 PMC11279761

[13] I. Needleman , R. Tucker , E. Giedrys‐Leeper , and H. Worthington , “Guided Tissue Regeneration for Periodontal Intrabony Defects–a Cochrane Systematic Review,” Periodontology 2000 37, no. 1 (2005): 106–123, 10.1111/j.1600-0757.2004.37101.x. 15655028

[14] M. E. Peacock , D. A. Mott , M. F. Cuenin , et al., “Periodontal Plastic Surgical Technique for Gingival Fenestration Closure,” General Dentistry 49, no. 4 (2001): 393–395. 12016683

[15] J. T. Mellonig , “Decalcified Freeze‐Dried Bone Allograft as an Implant Material in Human Periodontal Defects,” International Journal of Periodontics and Restorative Dentistry 4, no. 6 (1984): 40–55. 6396269

[16] T. F. Flemmig , B. Ehmke , K. Bolz , et al., “Long‐Term Maintenance of Alveolar Bone Gain After Implantation of Autolyzed, Antigen‐Extracted, Allogenic Bone in Periodontal Intraosseous Defects,” Journal of Periodontology 69, no. 1 (1998): 47–53, 10.1902/jop.1998.69.1.47. 9527561

[17] G. Pecora , S. Kim , R. Celletti , and M. Davarpanah , “The Guided Tissue Regeneration Principle in Endodontic Surgery: One‐Year Postoperative Results of Large Periapical Lesions,” International Endodontic Journal 28, no. 1 (1995): 41–46, 10.1111/j.1365-2591.1995.tb00155.x. 7642328

[18] R. Travassos , B. Soares , S. H. Bhandi , et al., “Multidisciplinary Treatment of a Fenestration‐Type Defect,” Journal of Contemporary Dental Practice 16, no. 4 (2015): 329–334, 10.5005/jp-journals-10024-1685. 26067739

[19] M. P. Rajula , K. Varatharajan , R. Mani , and S. Krishnakumar , “Gingival Fenestration Management: A Rarefied Case Entity and Literature Review,” Journal of Pharmacy & Bioallied Sciences 12, no. Suppl 1 (2020): S648–S651, 10.4103/jpbs.JPBS_77_20. 33149537 PMC7595449

[20] S. Balasubramanian , V. Singh , S. Bhat , et al., “Isolated Mucosal Fenestration With Localized Gingival Recession: Closure With an Acellular Dermal Graft. A Rare Case Report With Two Years' Follow‐Up,” Quintessence International (Berlin, Germany: 1985) 47, no. 5 (2016): 425–431, 10.3290/j.qi.a35522. 26824083

[21] V. Chandra , V. K. Bains , R. Jhingran , R. Srivastava , and R. Madan , “Comparative Evaluation of Platelet‐Rich Fibrin Versus Connective Tissue Grafting in Treatment of Gingival Recession Using Pouch and Tunnel Technique: A Randomized Clinical Study,” Contemporary Clinical Dentistry 13, no. 3 (2022): 217–226, 10.4103/ccd.ccd_749_20. 36213855 PMC9533392

[22] J. Choukroun , A. Diss , A. Simonpieri , et al., “Platelet‐Rich Fibrin (PRF): A Second‐Generation Platelet Concentrate. Part V: Histologic Evaluations of PRF Effects on Bone Allograft Maturation in Sinus Lift,” Oral Surgery, Oral Medicine, Oral Pathology, Oral Radiology, and Endodontics 101, no. 3 (2006): 299–303, 10.1016/j.tripleo.2005.07.012. 16504861

[23] V. R. Kumar and G. Gangadharan , “Platelet Rich Fibrin in Dentistry: A Review of Literature,” International Journal of Medicine 3, no. 2 (2015): 72–76, 10.14419/ijm.v3i2.5079.

[24] J. S. Patel , S. G. Patel , and C. Kadam , “Choukroun's Platelet Rich Fibrin in Regenerative Dentistry,” Universal Research Journal of Dentistry 3, no. 1 (2013): 22–25, 10.4103/2249-9725.119046.

[25] S. Friedman , “Retrograde Approaches in Endodontic Therapy,” Dental Traumatology 7, no. 3 (1991): 97–107, 10.1111/j.1600-9657.1991.tb00192.x. 1782906

[26] F. Gerhards and W. Wagner , “Sealing Ability of Five Different Retrograde Filling Materials,” Journal of Endodontia 22, no. 9 (1996): 463–466, 10.1016/S0099-2399(96)80078-1. 9198426

[27] T. Von Arx , M. Penarrocha , and S. Jensen , “Prognostic Factors in Apical Surgery With Root‐End Filling: A Meta‐Analysis,” Journal of Endodontia 36, no. 6 (2010): 957–973, 10.1016/j.joen.2010.02.026. 20478447

[28] I. Abramovitz , H. Better , A. Shacham , et al., “Case Selection for Apical Surgery: A Retrospective Evaluation of Associated Factors and Rational,” Journal of Endodontia 28, no. 7 (2002): 527–530, 10.1097/00004770-200207000-00010. 12126382

